# Recurrent dermatofibrosarcoma protuberans of the anterior abdominal wall with challenging post-excision anterior wall reconstruction: case report and review of the literature

**DOI:** 10.1093/jscr/rjaf845

**Published:** 2026-04-22

**Authors:** Oshiozimede Quincy Aigbonoga, Oluwafemi Awe, Orume Enegbuya, Andrew Akarutu Okomayin, Johnbull Mazor Akerele, Stella Nneka Ngwu, Ewoigbe Ikhuoria, Daniel Ehileboh, Charles Ikhifa, Fidelis Ozoba, Miracle Onosetale Edokpa, Unity Akugbe Ehijie

**Affiliations:** Plastic and Reconstructive Unit, Department of Surgery, Irrua Specialist Teaching Hospital, Km 87 Benin-Auci Expressway, Irrua, 310112, Nigeria; ADIZA Hospital, Km 135 Benin-Okene-Abuja Expressway, Jattu, 312102, Nigeria; Plastic and Reconstructive Unit, Department of Surgery, Irrua Specialist Teaching Hospital, Km 87 Benin-Auci Expressway, Irrua, 310112, Nigeria; Department of Anatomic Pathology, Irrua Specialist Teaching Hospital, Km 87 Benin-Auchi Expressway, Irrua, 310112, Nigeria; Department of Surgery, Irrua Specialist Teaching Hospital, Km 87 Benin-Auci Expressway, Irrua, 310112, Nigeria; Department of Surgery, Irrua Specialist Teaching Hospital, Km 87 Benin-Auci Expressway, Irrua, 310112, Nigeria; Department of Surgery, Irrua Specialist Teaching Hospital, Km 87 Benin-Auci Expressway, Irrua, 310112, Nigeria; Department of Surgery, Irrua Specialist Teaching Hospital, Km 87 Benin-Auci Expressway, Irrua, 310112, Nigeria; Department of Surgery, Irrua Specialist Teaching Hospital, Km 87 Benin-Auci Expressway, Irrua, 310112, Nigeria; Department of Anaesthesiology, Irrua Specialist Teaching Hospital, Km 87 Benin-Auci Expressway, Irrua, 310112, Nigeria; Plastic and Reconstructive Surgery Department, University of Benin Teaching Hospital, Ugbowo-Lagos Road, Benin, 300001, Nigeria; Department of Surgery, Asaba Specialist Hospital, Mid-Wifery Road, Off Akpanam Road, Asaba, 320211, Nigeria; ADIZA Hospital, Km 135 Benin-Okene-Abuja Expressway, Jattu, 312102, Nigeria

**Keywords:** dermatofibrosarcoma protuberans, huge anterior abdominal wall mass, multilayered anterior abdominal wall involvement, anterior abdominal wall reconstruction

## Abstract

Dermatofibrosarcoma protuberans is an uncommon low-grade soft tissue tumour with a reasonably high recurrence rate. It represents an indolent, locally aggressive soft tissue tumour that arises from the dermal and subcutaneous layers. Large anterior abdominal wall dermatofibrosarcoma provides a unique challenge in its surgical oncology management due to its potential considerable size at the time of presentation and diagnosis, ability to invade into critical adjoining structures, and residual normal tissues available for reconstruction. This publication article presents a case report of a patient with recurrent locally aggressive dermatofibrosarcoma of the anterior abdominal wall who had a wide local excision biopsy with resultant extensive full-thickness anterior abdominal wall defect that was reconstructed using bilateral rotational transversus abdominis muscle flap and polypropylene meshplasty. Provided also in the article is the detailed review of the clinical presentation, radiological findings, surgical procedure, adjuvant therapy, and the outcome of management.

## Introduction

Soft tissue sarcomas are mesenchymal neoplasms comprising of adult malignant tumours. Among soft tissue tumours, abdominal wall tumours are uncommon accounting for less than 5% of these neoplasms [1]. The most common soft tissue tumour of the abdominal wall is the desmoid tumour while the least frequent of them is the dermatofibrosarcoma protuberans (DFSP) which accounts for 1%–6% of soft tissue sarcomas, and 18% of cutaneous soft tissue sarcomas [1–3]. They are commonly found on the trunk and extremities [1].

## Case report

**Figure 1:**
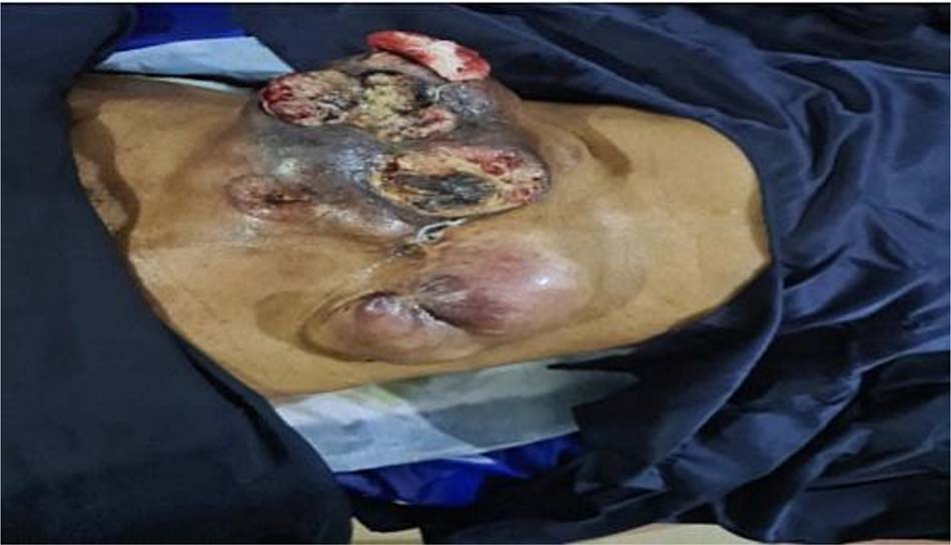
Preoperative photograph showing multinodular, ulcerated mass with shiny telangiectatic perilesional skin.

**Figure 2:**
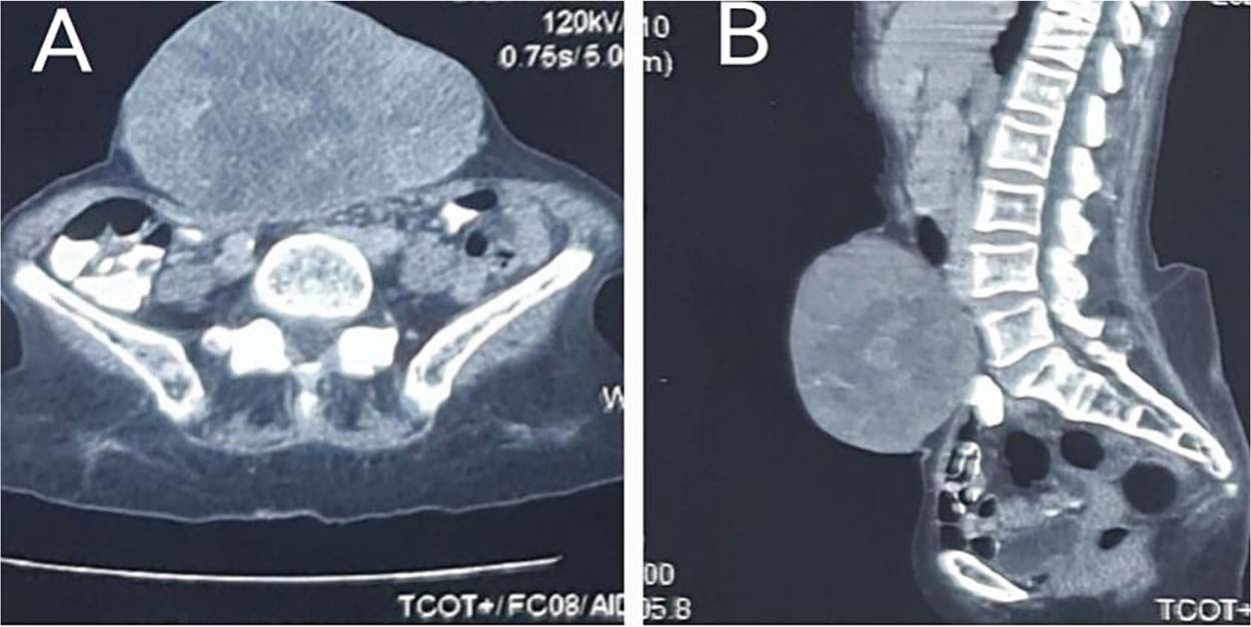
Axial (A) and sagittal (B) planes showing invasion of the anterior abdominal muscles and fasciae.

**Figure 3:**
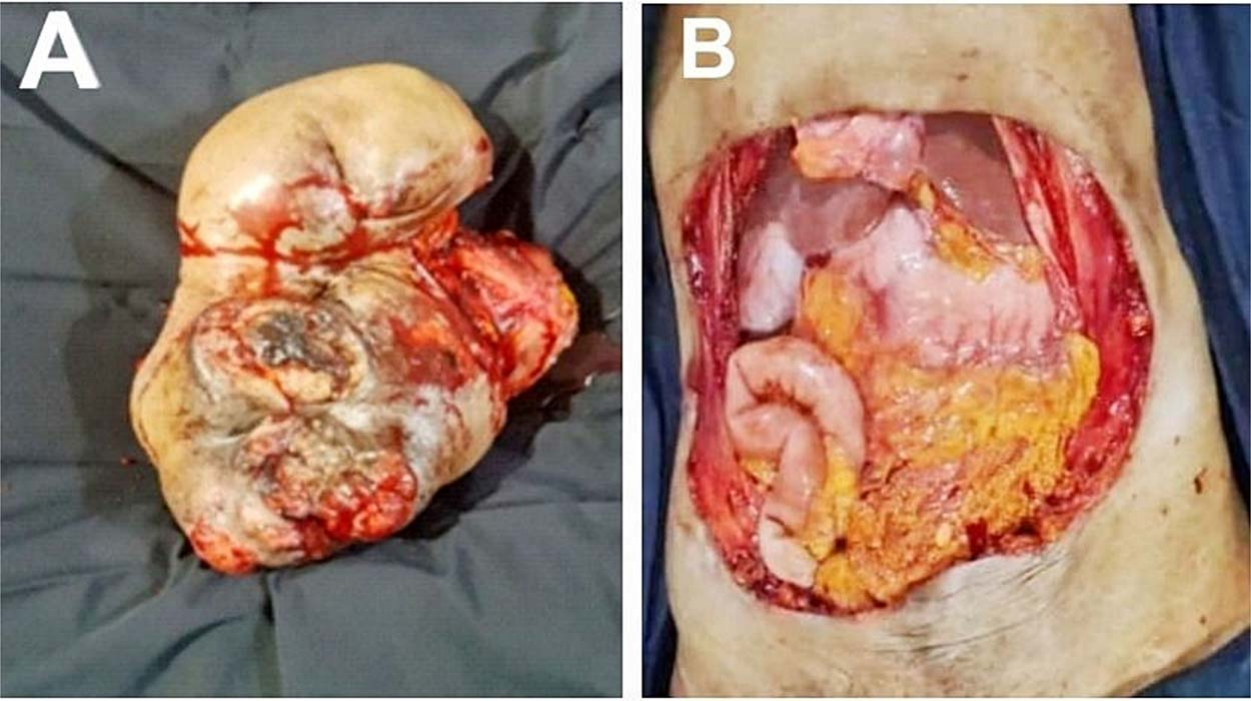
Excised full-thickness anterior abdominal mass (A). Post-resection anterior abdominal defect (B).

A 66-year-old woman presented with a recurrent slow-growing tumour on the anterior abdominal wall, following three separate surgical resections over a 21-year period. The present tumour occurred 5 years prior to presentation. Examination revealed an extensive multinodular anterior abdominal wall mass measuring 32 × 25 cm, with ulcerations at the nodules, surrounded by shiny perilesional telangiectatic skin (Fig. 1). Abdominal computed tomography (CT) scan revealed a well-circumscribed, multinodular mass adherent to the overlying skin, with infiltration into the subcutaneous tissue, recti sheaths and muscles, and other abdominal wall muscles (Fig. 2A and B). A wide local excision (WLE) of the tumour (1.6 kg in weight) (Fig. 3A) with a circumferential 3 cm macroscopic tumour-free margin was performed (Fig. 3B).

**Figure 4:**
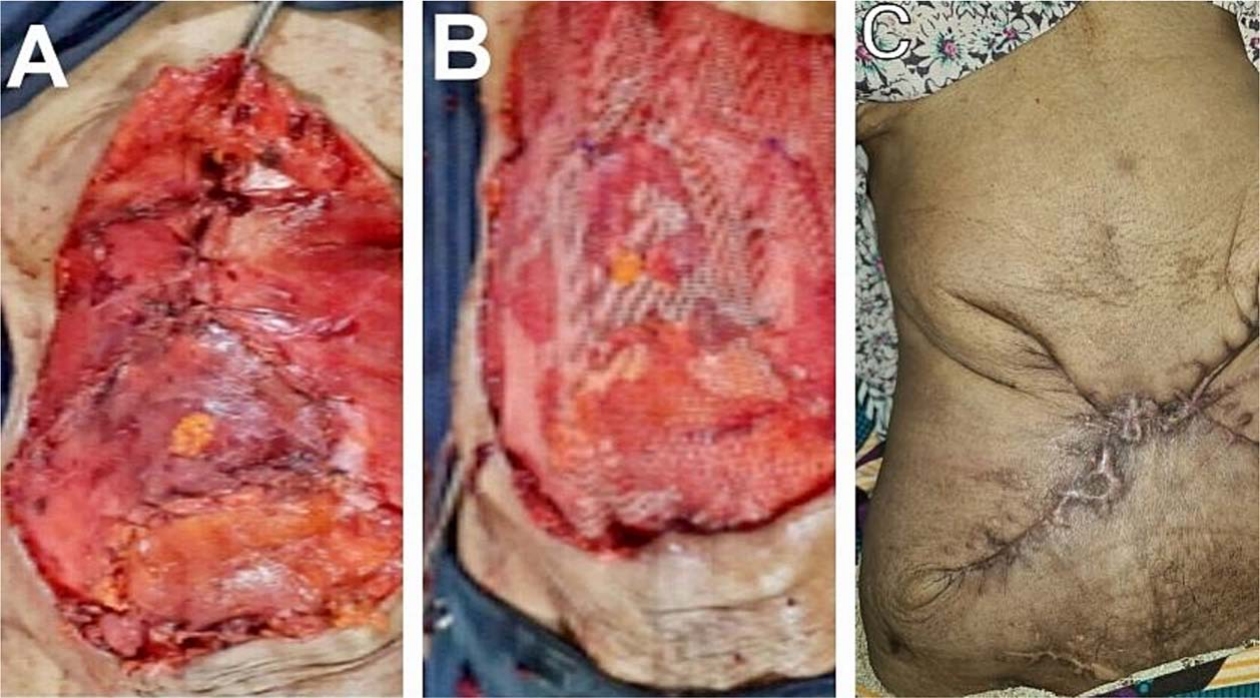
Rotational advancement external oblique muscle flaps (A). Onlay polypropylene mesh reinforcement (B). Nine-month appearance of skin closure in an X-plasty fashion (C).

**Figure 5:**
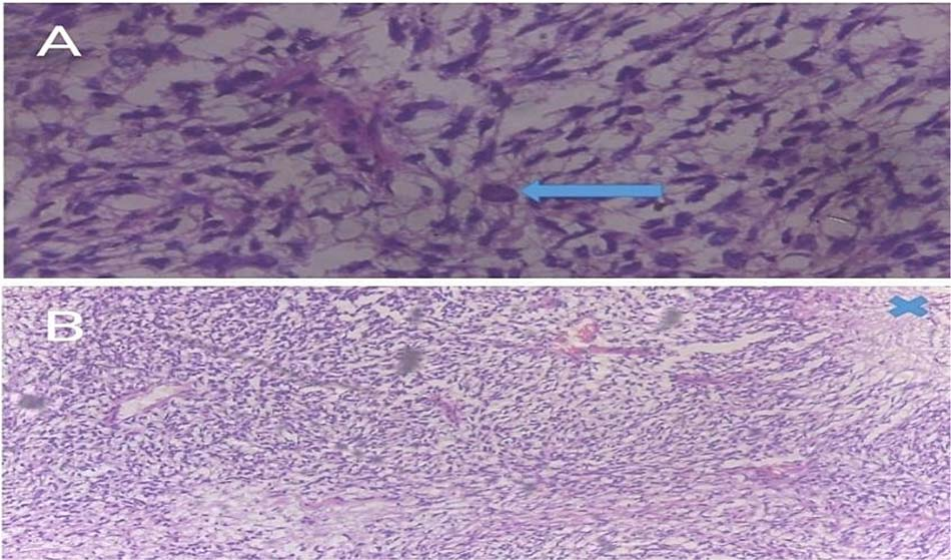
(A) A 400× photomicrograph of a haematoxylin and eosin (H&E)-stained slide showing spindle-shaped cells in a loose fibromyxoid matrix indicating a fibrosarcomatous transformation. A bizarre cell (arrow) is seen in the centre. (B) Spindle-shaped cells in a whorled or cart-wheel pattern with areas of cell necrosis (marked X).

The post-resection full-thickness defect measured 38 × 30 cm and was reconstructed using bilateral inferiorly based external oblique muscles flaps with onlay polypropylene meshplasty. The mobilized muscle flaps on both sides of the anterior abdominal wall defect were then rotated medially and sutured to each other at the midline (Fig. 4A). An onlay polypropylene mesh reinforcement was then performed, anchored in place using Prolene 3/0 suture in a simple interrupted fashion (Fig. 4B). A subcutaneous negative-pressure suction drain was inserted (which was discontinued on the postoperative day seven), and the skin closure was performed in an O-X plasty with a minimal tension-free pattern using Prolene 2/0 suture in a simple interrupted fashion (Fig. 4C). Histopathological sections showed a malignant mesenchymal tumour composed of alternating hypercellular and hypocellular areas with perivascular accentuation (Fig. 5). The cells are spindle-shaped with hyperchromatic thin wavy nuclei and scanty cytoplasm disposed within scanty fibroconnective stroma having vague herring-bone patterns, necrosis, and congested vessels. This finding was in keeping with fibrosarcomatous changes of the tumour.

The postoperative period was uneventful. The patient was commenced on daily adjuvant imatinib and has been tumour-free for the last nine (9) months postoperative period.

## Discussion

DFSP is an uncommon tumour that originates from the dermal, subcutaneous, and in exceptional cases, muscles, fasciae, and bone [4]. However, it is the most common skin sarcoma [5]. It is a superficial slow-growing, locally aggressive infiltrative fibroblastic dermal sarcoma of low- to intermediate-malignancy with high recurrence and low metastatic potential [3, 4]. In 5%–20% of cases, there are high-grade fibrosarcomatous components, and this transformation is responsible for high incidence of local relapse and distant metastases [6, 7]. However, there was no evidence of metastasis. The most common predisposing factor is recurrent trauma to the skin [3, 4]. Also, preexisting cutaneous scars and tattoos may increase the risk of developing the tumour [4]. Its most frequently affected location is the trunk, extremities, and then the head and neck [5]. Its annual incidence is 0.8%–4.5% worldwide [6]. It accounts for less than 5% of soft tissue tumours and 0.1% of malignancies [6]. The pathogenesis remains unclear, although 10%–20% of patients report a history of trauma. In 80%–90% of cases, there is a somatic mutation involving the proto-oncogene platelet-derived growth factor beta polypeptide gene (PDGFβ) and the collagen type 1A1 gene (COL1A1) [2, 3]. This is formed by translocation of 17 and 22 chromosomes (17:22), which causes activation of the tyrosine-kinase pathway leading to PDGFβ overexpression [2, 3].

Fibrosarcomatous transformation in a classical DFSP occurs in 5%–20% of cases [7]. It is an aggressive variant with high rate of local recurrence and high distant metastatic potential. The features that are suggestive of fibrosarcomatous transformation within DFSP are at least 5% area of spindle cells, mitotic index seven-tenths (7/10) high power field, high pleomorphism, and herringbone pattern [7]. The abdominal localization of DFSP remains among the rare malignant tumours of the soft tissues of the abdomen which are dominated by the desmoid tumours [8]. CT scan or magnetic resonance imaging has been used to stage these abdominal tumours [1].

Surgical excision with clear margins is the primary treatment for DFSP [4]. WLE biopsy with a 2 cm margin of healthy tissue, and MOHS micrographic surgery (where facilities are available) is often recommended [4]. Resection with 2–4 cm margin has a 20% recurrence rate due to eccentric growth in the form of ‘tentacles or pseudopods’ [9].

Reconstruction of the abdominal defect after DFSP excision poses a unique challenge, as it requires both functional and aesthetic restoration [4]. Available reconstructive options for the post-excision anterior abdominal defect exist, and they include direct repair, skin grafts, local flaps, regional flaps, free fascial graft, component separation, tissue expansion, vacuum-assisted closure device, free flaps, and prosthetic mesh repair [4, 9]. In the reported case, the post-surgical anterior abdominal wall defect was reconstructed using bilateral inferiorly based rotational advancement external oblique flaps with an onlay polypropylene mesh reinforcement technique. This is a single-stage minimal tension, primarily functional, aesthetic and cost-effective surgical procedure that was customized to the patient’s post-surgical anterior abdominal wall defect.

The chemotherapeutic agent imatinib mesylate is usually restricted for recurrent, unresectable, or metastatic cases in adults [2]. It competitively inhibits binding of PDGFβ-receptor [2]. The usual dose of imatinib is 400 mg/day and in cases with poor response, the dose can be increased to 600–800 mg/day [7]. The response rate of imatinib is about 65% [2]. Adjuvant radiotherapy has been claimed to be controversial [1]. However, some authorities believe that it should be indicated for unresectable, or recurrent tumours [2]. Prognosis of anterior abdominal DFSP is defined by the grade of the tumour which is measured by the mitotic activity. Patients with age >50 years, resection margin <2 cm, and fibrosarcomatous variant have poor prognosis. Tumours with high Ki 67 show poor prognosis [7].

## Conclusion

Anterior abdominal wall DFSP is an indolent, highly recurrent sarcoma. The fundamental pillar of treatment consists of a multidisciplinary approach (oncologist, oncological surgeons, dermatologists, pathologists, and plastic surgeons. Adequate resection margin with dynamic composite anterior abdominal wall reconstruction and adjuvant imatinib mesylate gives a good prognosis.
